# Reliability and clinical applicability of a novel tear film imaging tool

**DOI:** 10.1007/s00417-021-05162-8

**Published:** 2021-03-29

**Authors:** Noémi Tóth, Eszter Szalai, Tibor Rák, Veronika Lillik, Attila Nagy, Adrienne Csutak

**Affiliations:** 1 Department of Ophthalmology, University of Pécs, Medical School, Akác u. 1, Pécs, 7623 Hungary; 2 Doctoral School of Clinical Medicine, University of Debrecen, Debrecen, 4032 Hungary; 3 Medical School, University of Pécs, Pécs, 7624 Hungary; 4 Department of Preventive Medicine, Faculty of Public Health, University of Debrecen, Debrecen, 4028 Hungary

**Keywords:** Dry eye disease, Lower tear meniscus height, Tear film break-up time, Meibography, Interferometry

## Abstract

**Purpose:**

The aim of our research was to investigate the reliability and clinical applicability of a modern tear film imaging tool by comparing the inter- and intragrader difference. The further goal was to compare the non-invasive tear break-up time (NIBUT) measured with the LacryDiag® device with traditional tear film break-up time (TBUT).

**Methods:**

Comprehensive ophthalmological examination was performed, including LacryDiag® (Quantel Medical, France) (lower tear meniscus height measuring (LTMH), superior and inferior eyelid meibography (MeibS MeibI), interferometry (INT), NIBUT), slit lamp examination, and TBUT. Two independent, well-trained graders selected and analyzed the LTMH, MeibI, MeibS, and INT. The second grader reanalyzed the data 1 month later. Intra- and inter-examiner reliabilities were evaluated using intraclass correlation coefficients (ICC), while for categorical variable, Cohen’s kappa statistics were provided. The Bland-Altman plot was used for visualization of the agreement between measurements.

**Results:**

Fifty healthy volunteers were examined. For LTMH both the inter- and intragrader variabilities were excellent. Between two graders, the ICC of MeibI was poor; however, between two graders, the ICC of MeibS was good, and the intragrader variability in MeibI and MeibS was excellent. For the INT, both intra- and intergrading were in fair and moderate agreement, although the intragrader agreement was higher. Comparing the NIBUT and TBUT, the agreement was slight.

**Conclusion:**

Based on our results, examination of a patient during follow-up should be performed by the same examiner, because of the slight agreement. The LacryDiag® is a non-invasive, easy-to-use device, which can examine the tear film and save the recordings for easier follow-up.

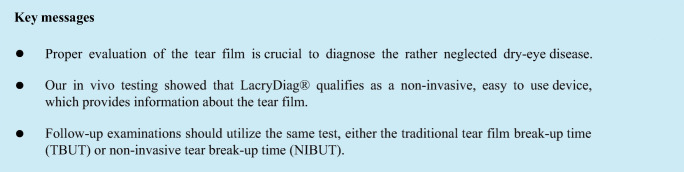



## Introduction

Dry eye disease (DED) is a common ocular disorder occurring worldwide. It is characterized by tear film instability and hyperosmolarity, inflammation, and consequent damage to the ocular surface. The 2017 Dry Eye Work Shop (DEWS) II report provided the following DED definition: “Dry eye is a multifactorial ocular surface disease characterized by loss of tear film homeostasis associated with ocular symptoms, in which tear film instability and hyperosmolarity, inflammation and ocular surface lesions, as well as neurosensory abnormalities play etiologic roles” [1]. The global prevalence in the adult population ranges from 5–7% in the USA to 30–50% in the Far East and Africa [2]. The prevalence in Europe lies in the middle of the range [3–6]. Concomitant signs of a dry eye (redness, burning sensation, photosensitivity, and excessive lacrimation upon external effect) are one of the most frequent reasons for patients to see an eye care practitioner. The disease mostly affects the middle-aged and older population, but the incidence is rising among the youth [1, 7, 8]. As a consequence, clear vision is compromised, quality of life is decreasing, and work productivity is declining [7, 9]. For these reasons, it is crucial for ophthalmologists to recognize the signs of DED by applying reliable diagnostic methods.

The diagnosis of the worldwide extremely common DED is based on the subjective complaints which can be quantified by the ocular surface–disease index (OSDI) questionnaire and is confirmed by examination with slit and focal light. Further diagnostic tests include Schirmer test; tear film break-up time (BUT); staining with fluorescein, bengal rose, and lissamine green; crystallization test; tear film osmolarity; and semiquantitative tear film interferometry. These methods can detect changes in the quality and quantity of components of tear film [10].

**Fig. 1:**
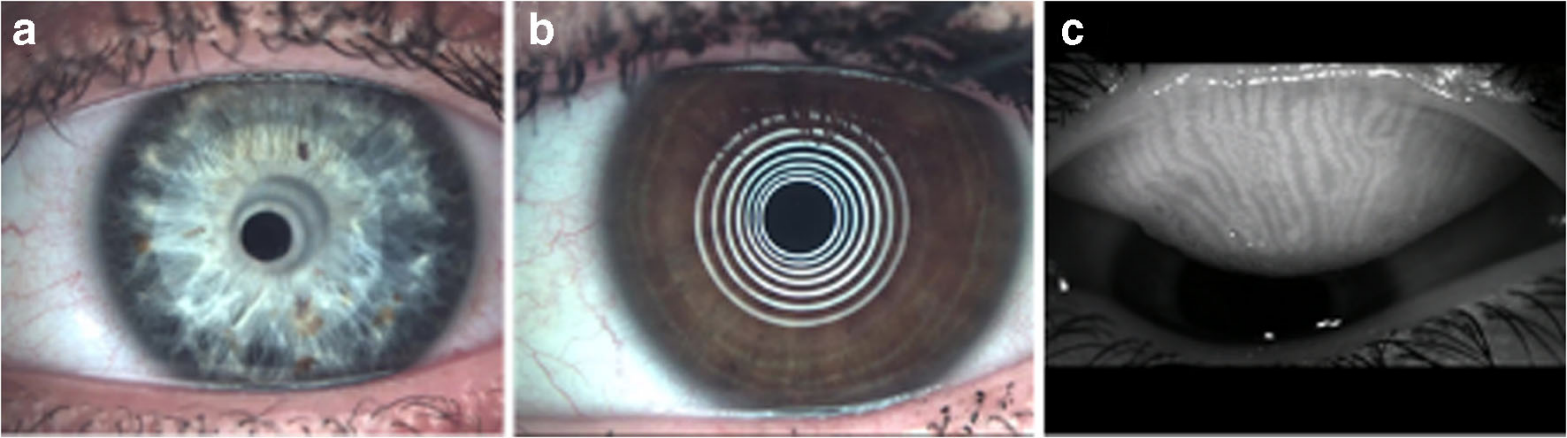
Interferometry, lower tear meniscus height (**a**), non-invasive tear break-up time (**b**), and upper eyelid photo for meibography (**c**) with LacryDiag® device (own source)

The novel LacryDiag® (Quantel Medical, France) device can diagnose dry eye disease with numerous non-contact exams, such as measuring the lower tear meniscus height (LTMH), superior (MeibS) and inferior (MeibI) eyelid meibography, tear interferometry (INT), and non-invasive tear film break-up time (NIBUT) (Fig. 1). There are some additional exams that can be used for dry eye diagnosis, e.g., blepharitis and demodex imaging, bulbar redness, staining (corneal, conjunctiva, and lid margin), pupillometry, white to white measurement, and corneal deformation. LTMH, MeibS, MeibI, INT. and NIBUT comply with the criteria of the TFOS DEWS 2 report; hence, these non-contact examinations were evaluated in this study [11].

The aim of our research was to investigate the reliability and clinical applicability of a modern tear film imaging tool by comparing the inter- and intragrader difference. A further goal was to investigate if modern diagnostic tools are different from conventional reference methods; hence, the NIBUT measured with the LacryDiag® device was compared to TBUT.

## Material and methods

### Study participants

Fifty healthy volunteers were enrolled in this study from the Department of Ophthalmology, University of Pécs, Medical School, in accordance with the Declaration of Helsinki. The study was approved by the Institutional Ethics Committee. Subjects had no past or current history of any general or ocular diseases, and no participant had a history of contact lens wear intraocular or any type of refractive surgery. The mean age of volunteers was 27.15±1.36 years; their gender was 20 male and 30 female.

### Ophthalmological examination

Comprehensive ophthalmological examination was carried out, including LacryDiag® (Quantel Medical, France), slit lamp examination, and TBUT. The TBUT was performed at least 5 min after the LacryDiag® examination [12].

The LacryDiag® examination and TBUT measurement were performed by one well-trained examiner. Two independent, well-trained graders selected and analyzed the LTMH, the MeibS, the MeibI, and the INT. The second grader reanalyzed the data 1 month later [13].

The procedure took approximately 10 min to perform, and none of the subjects complained of pain, inconvenience, or visual disturbance.

Measuring the height of the tear meniscus gives a quantitative assessment of the aqueous phase. The majority of tear is comprised within the menisci; this is the margin part of the upper and lower eyelids meeting the bulbar conjunctiva [14]. The quantitative analysis of the tear menisci is the most direct approach to study the volume of the tear film [6, 7]. The LacryDiag® device performs the measurement of LTMH semi-automatically with two calipers placed by the observer on the lacrimal river. The average of five measurements was used for the analysis.

During meibography, the silhouette of the glands on the inner surface of the eyelids is visualized and analyzed by selecting a given area to calculate the percentage of meibomian glands loss. The technique is based on white-light transillumination of everted eyelids from the skin aspect [15]. With this semi-automatic method, the search area is drawn by the examiner, and the meibomian glands loss area is given by the device.

Tear interferometry is a qualitative and quantitative analysis of the lipid layer. Oily substances spreading in a thin layer on the surface of water can be detected by this method [15]. Based on the reflection pattern and kinetics of the oily phase, the thickness of lipid layer could be evaluated. This component of tear film is mostly produced by the meibomian glands [16]. Interferometry is measured with LacryDiag® device comparing the actual patient’s video recording with a grading scale (seven of predefined videos).

Since it is well known that tear film stability can be affected by many external factors, NIBUT has become more and more commonly applied [15, 17]. The NIBUT determines tear film stability by means of the extent of evaporation. This method is based on observation of the reflection of an illuminated grid pattern from the tear film [18]. NIBUT software automatically detects blinks, records the interblink interval, and calculates the NIBUT result. According to the manual, if the interblink interval reaches 12 s, the recording should be stopped. Since the examiner’s task is limited to starting and stopping the recording and does not involve grading, there was no need to compare inter- or intragrader agreement. Instead, NIBUT was compared with standard TBUT. Reference values of NIBUT and TBUT used a cut-off time of 10 s [19–24]; however, 12-s cut-off at NIBUT and 8-s cut-off at TBUT showed better diagnostic ability [20]. Cut-off times of 10 s for both NIBUT and TBUT, as well as 12 s for NIBUT and 8 s for TBUT, were used here. For statistical analysis, an ordinal scale was used to decrease statistical aberration.

### Statistical analysis

Statistical analysis was carried out using Intercooled Stata for Windows (version 13.0). The data of the right eyes were used. Intraclass correlation coefficients (ICC)—consistency (without cut-off value)—and their 95% confidence intervals (CI) were also provided to estimate intra- and inter-examiner reliability at LTMH, MeibS, and MeibI. An ICC below 0.4 indicates poor reliability, an ICC between 0.4 and 0.59 indicates fair reliability, an ICC between 0.6 and 0.74 indicates good reliability, and an ICC between 0.75 and 1.0 indicates excellent reliability [25]. To assess the agreement in INT, and between NIBUT and TBUT, weighted Cohen’s kappa statistic was used as they had ordinal categories. The interpretation of Cohen’s kappa under 0.2 indicates slight agreement, 0.21 to 0.40 indicates fair agreement, 0.41–0.60 indicates moderate agreement, 0.61–0.80 indicates substantial agreement, and 0.81 to 1.0 indicates almost perfect agreement [26]. The Bland-Altman plot was used for visualization of the agreement between measurements, and 95% limits of agreement (LoA) was also calculated as mean±1.95 standard deviation (SD) of the difference. In these plots, a medium line indicates the mean difference between the devices, and the upper and lower lines show the 95% LoA values [27]. A *p*-value < 0.05 was considered statistically significant.

## Results

The examined fifty healthy volunteers’ descriptive data are shown in Table 1. Measuring TBUT, 15.6% of the volunteers were below 8 s of TBUT, and 28.12% of the volunteers were under 10 s of TBUT. Measuring NIBUT, 28.12% of the study subjects were under 10 s of NIBUT, and 75% of them were below 12 s of NIBUT.

**Table 1:** Descriptive statistic of LacryDiag® examination

		Tear meniscus height (mm)		
		I.	II.	III.
Mean ± SD		0.19 ± 0.01	0.21 ± 0.01	0.20 ± 0.01
95% CI	Lower bound	0.17	0.18	0.18
	Upper bound	0.22	0.23	0.22
		Interferometry		
		I.	II.	III.
Mean ± SD		2.82 ± 0.18	3.04 ± 0.14	2.92 ± 0.16
95% CI	Lower bound	2.45	2.76	2.59
	Upper bound	3.18	3.32	3.24
		Inferior meibography (%)		
		I.	II.	III.
Mean ± SD		18.81 ± 2.00	29.63 ± 1.38	31.43 ± 1.34
95% CI	Lower bound	14.78	26.90	28.73
	Upper bound	22.85	32.45	34.13
		Superior meibography (%)		
		I.	II.	III.
Mean ± SD		6.54 ± 1.30	19.69 ± 1.52	21.41 ± 1.44
95% CI	Lower bound	3.92	16.62	18.51
	Upper bound	9.17	22.76	24.31

I: Grader 1

II: Grader 2 1st grading

III. Grader 2 2nd grading

*CI* confidence interval

*SD* standard deviation

**Fig. 2:**
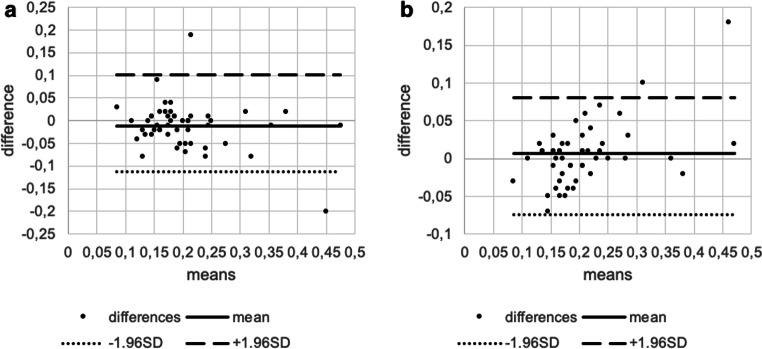
Bland-Altman plots showing the agreement between inter- (**a**) and intragraders (**b**) measuring low tear meniscus height. The dots represent the differences from the mean value, the continuous line illustrates the mean value, the dotted line depicts the −1.96 standard deviation (SD), and the broken line is for +1.96 SD. Intercooled Stata for Windows (version 13.0) and Microsoft Excel (version 16.0) were used to create the figure

**Fig. 3:**
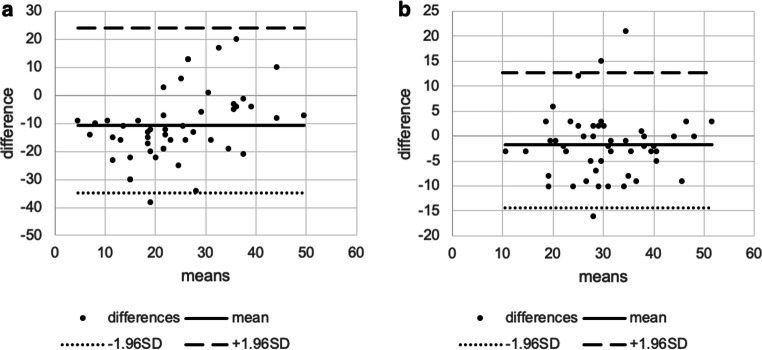
Bland-Altman plots showing the agreement between inter- (**a**) and intragraders (**b**) measuring Meibomian gland loss in the lower eyelid. The dots represent the differences from the mean value, the continuous line illustrates the mean value, the dotted line depicts the −1.96 standard deviation (SD), and the broken line is for +1.96 SD. Intercooled Stata for Windows (version 13.0) and Microsoft Excel (version 16.0) were used to create the figure

**Fig. 4:**
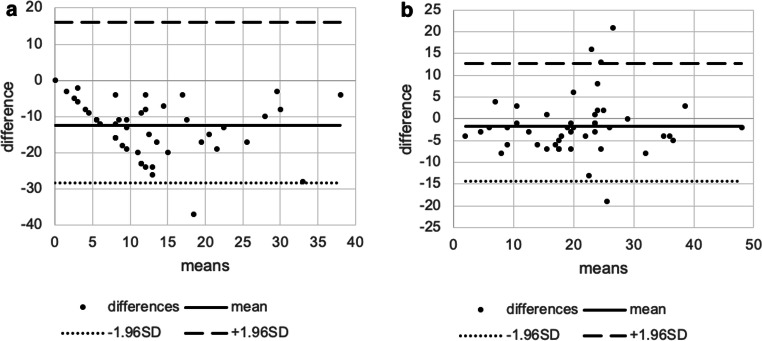
Bland-Altman plots showing the agreement between inter- (**a**) and intragraders (**b**) measuring Meibomian gland loss in the upper eyelid. The dots represent the differences from the mean value, the continuous line illustrates the mean value, the dotted line depicts the −1.96 standard deviation (SD), and the broken line is for +1.96 SD. Intercooled Stata for Windows (version 13.0) and Microsoft Excel (version 16.0) were used to create the figure

For LTMH both the inter- and intragrader variabilities were excellent (intergrader ICC = 0.805, intragrader ICC= 0.868). Between two graders, the ICC of MeibI was poor (MeibI ICC=0.464); however, between two graders, the ICC of MeibS was good (MeibS ICC=0.666), and the intragrader variability in MeibI and MeibS were excellent (MeibI ICC=0.760; MeibS ICC=0.771) (all *p* values were <0.001) (Table 2). The Bland-Altman plots for LTMH, MeibI, and MeibS showed high variability of LoA for all parameters between the groups (Table 2; Figs. 2, 3, and 4).

**Table 2:** Inter- and intragrader variability in lower tear meniscus height and lower and upper eyelid meibography

Tear meniscus height (mm)			
Intergrader variability	Mean intergrader difference		−0.011
	95% LoA		−0.11 to 0.101
	ICC		0.805
	95% confidence interval	Lower bound	0.68
		Upper bound	0.884
	*p* value		<0.001
Intragrader variability	Mean intragrader difference		0.006
	95% LoA		−0.07 to 0.08
	ICC		0.868
	95% confidence interval	Lower bound	0.778
		Upper bound	0.923
	*p* value		<0.001
Inferior meibography (%)			
Intergrader variability	Mean intergrader difference		−10.64
	95% LoA		−34.78 to 24.14
	ICC		0.464
	95% confidence interval	Lower bound	0.213
		Upper bound	0.658
	*p* value		<0.001
Intragrader variability	Mean intragrader difference		−1.72
	95% LoA		−14.40 to 12.68
	ICC		0.76
	95% confidence interval	Lower bound	0.610
		Upper bound	0.857
	*p* value		<0.001
Superior meibography (%)			
Intergrader variability	Mean intergrader difference		−12.35
	95% LoA		−28.35 to 16.01
	ICC		0.666
	95% confidence interval	Lower bound	0.467
		Upper bound	0.800
	*p* value		<0.001
Intragrader variability	Mean intragrader difference		−1.61
	95% LoA		−14.40 to 12.79
	ICC		0.77
	95% confidence interval	Lower bound	0.622
		Upper bound	0.867
	*p* value		<0.001

*LoA* limits of agreement, *ICC* intraclass correlation coefficient

**Fig. 5:**
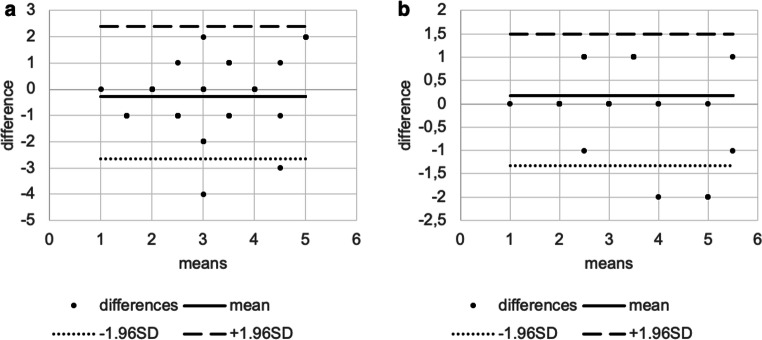
Bland-Altman plots showing the agreement between inter- (**a**) and intragraders (**b**) examining interferometry. The dots represent the differences from the mean value, the continuous line illustrates the mean value, the dotted line depicts the −1.96 standard deviation (SD), and the broken line is for +1.96 SD. Intercooled Stata for Windows (version 13.0) and Microsoft Excel (version 16.0) were used to create the figure

Grading the INT, both intra- and intergrading were in fair and moderate agreement (INT intergrader value=0.301; *p*=0.0002; INT intragrader value=0.566; *p*<0.001), although the intragrader agreement was higher (Table 3). The Bland-Altman plots for INT showed high variability of LoA for all parameters between the groups (Table 3; Fig. 5).

**Table 3:** Inter- and intragrader reliability in interferometry, comparing TBUT with NIBUT

Interferometry	Intergrader reliability	Mean intergrader difference	−0.28
		95% LoA	−2.66 to 2.38
		Kappa coefficient	0.301
		*p* value	0.0002
	Intragrader reliability	Mean intragrader difference	0.18
		95% LoA	−1.32 to 1.50
		Kappa coefficient	0.566
		*p* value	<0.001
Break-up time (sec)	NIBUT cut-off 12 sec, TBUT cut-off 8 sec	Kappa coefficient	0.075
		*p* value	0.099
	NIBUT and TBUT cut-off 10 sec	Kappa coefficient	0.054
		*p* value	0.376

*LoA* limits of agreement, *NIBUT* non-invasive tear break-up time, *TBUT* traditional tear break-up time

Comparing the NIBUT and TBUT, all agreements were slight (NIBUT cut-off 12 s, TBUT 8 s: kappa coefficient =0.075; *p*=0.099; NIBUT and TBUT cut-off 10 s: kappa coefficient =0.054; *p*=0.376) (Table 3).

## Discussion

The parameters analyzed in our study can be measured by several other methods. The advantage of LTMH is that the tear meniscus over the lower eyelid is readily visualized. The traditional way to measure LTMH is an examination with a slit lamp equipped with a micrometer scale; slit lamp photographs can be objectively analyzed.

Additional methods for measuring LTMH include optical coherence tomography (OCT), fundus camera, and Tearscope ® (Keeler, Windsor, UK). Fodor et al. found that there is no difference between the mean measured LTMH with Tearscope ®, slit lamp, and slit lamp with fluorescein, but the repeatability is better with the Tearscope ® device [28]. OCT is an imaging modality based on the quantity of reflected light from tissues; Wang et al. stated that OCT is a multifaceted technique to measure LTMH because it provides a real-time, non-invasive, high-quality image, although the mean value can be higher than that obtained by the traditional methods and the repeatability of the measurement is unfavorable [29]. Kawai et al. applied fundus camera to take pictures of the anterior surface of the eye. This method proved to be a useful and simple way to diagnose and follow-up DED because it measures not only the lower but the upper tear meniscus height too [30]. The LacryDiag® device evaluates LTMH semi-automatically based on the average of five estimations. In our study, this mechanism provides an excellent inter- and intragrader variability for LTMH. Therefore, this parameter can be used by any practitioner for follow-up, although the Bland-Altman plot showed high variability of LoA, which means disagreement between inter-and intragrader measurements.

Meibography is a useful tool when a Meibomian gland dysfunction is suspected, but to establish its diagnostic value, more accomplished studies are required [15]. Meibography should be used when accompanied by other parameters, e.g., interferometric values. There are various scoring scales, e.g., meiboscore, that can be practical and reliable in clinical practice because they are highly repeatable [31]. The traditional technique for meibography used white-light transillumination of the inner side of the eyelids and imaging based on black-and-white film, infrared film, or a near-infrared charge-coupled device (CCD) video camera. Arita et al. designed a non-invasive, slit lamp-based meibography system that involves an infrared filter and an infrared CCD video camera. This method provides faster imaging than other systems.

Technological advances facilitated meibography through the use of more modern, LED (light-emitting diode)-based, multifunctional devices connected to computers [15]. The LacryDiag® device uses an infrared imaging method of Meibomian glands and image analysis by automatic boundary detection and manual corrections if necessary. The color-coded graphic illustration allows fast interpretation of all four tests that can be implemented by LacryDiag®. In our study, the intergrader variable for MeibI was unsatisfactory; however, for MeibS it was fine. The intragrader variability for both MeibI and MeibS was great. The Bland-Altman plot showed high variability of LoA referring to poor agreement between inter-and intragrader measurements. As a consequence, this parameter should be assessed by the same practitioner for follow-up.

Interferometry is a popular diagnostic tool, because it is non-invasive, quick, and technician-friendly. Recently new devices were developed to measure INT, e.g., Tearscope Plus®, TearScience®, LipiView®. Tearscope Plus® uses wideband illumination to image the dynamics of the lipid layer of the tear film [15, 32]. LipiView® (TearScience Inc., Morrisville, NC) using Ocular Surface Interferometer (OSI) provides a great color presentation and image quality and has the potential to be an advantageous device in clinical practice [15, 33]. Goto et al. generated an algorithm for the DR-1 tear interference camera (Kowa, Nagoya, Japan) to measure lipid layer thickness from fringe patterns [15, 30]. Very recently, the lateral shearing interferometer has been proposed for research purposes; it uses fast Fourier transformation to analyze surface irregularities in the tear film [15]. The LacryDiag® device assesses interferometry by comparing patient’s recording with a grading scale of predefined videos. Calculating both intra- and intergrader variability, we can say that this tool provided decent agreement for INT; however, the value for intragrader variability was higher. On this account, the follow-up is more recommended to do by the same practitioner on one patient. The high variability of LoA based on the Bland-Altman plot also confirms this conclusion, because it means heterogeneity between inter- and intragrader measurements.

Although NIBUT is becoming increasingly applied, TBUT remains the most frequently employed test [15]. There are many ways to measure this parameter. Observation of reflection of an illuminated grid pattern from the tear film can be applied to measure NIBUT. More modern solutions involve image analysis of Placido’s disk with certain kinds of corneal topography systems attached to specific software. There are automated evaluation techniques of tear film stability, e.g., the Keratograph (Oculus, Wetzlar, Germany). This device detects and localizes tear break-up time with high-speed videokeratoscopy estimating the variance of the rings detected radially from the center of the image. It was further processed by Downie et al. using the E300 corneal topographer (Medmont International Pty Ltd., Victoria, Australia) to evaluate tear film surface quality break-up time with an algorithm that removes images with excessive movement and perceives shadows appearing because of the eyelashes [15, 17]. According to different studies, the values in these non-invasive methods better reflect tear film stability than those involving fluorescein [18, 34]. The LacryDiag® device applies automatic NIBUT measuring by analysis of reflected Placido disk images from the ocular surface. The NIBUT measurements by this modern tool led to slight agreement with the use of the traditional method; therefore, it is beneficial to use the same method for the follow-up of each patient.

The intragrader variabilities of LTMH, MeibI, and MeibS were excellent. The intergrader variability of LTMH was excellent, but the intergrader variability of MeibI and MeibS was poor and good, respectively. The agreement of INT examinations was fair (intergrader) and moderate (intragrader). Therefore, based on these results, the patients’ follow-up examinations for LTMH, MeibI, MeibS, and INT should be done by the same eye care practitioner. However, due to the slight agreement comparing NIBUT with TBUT, it is worthwhile to perform the follow-up using the same method.

There are some limitations in the present study. In the future, we plan to compare and analyze our results from the healthy subjects with DED patients, the real target group of this diagnostic tool. In addition, LacryDiag® tear diagnostic method comparisons should be organized with more intergrader observer examinations and increased healthy and DED subject numbers to draw stronger conclusions about this novel diagnostic device.

In conclusion, the LacryDiag® is a non-invasive, easy-to-use device, which can examine the tear film, with saving of the recordings for easier follow-up.

## Data Availability

Not applicable.
